# Preparation of Three-Dimensional MF/Ti_3_C_2_T_x_/PmPD by Interfacial Polymerization for Efficient Hexavalent Chromium Removal

**DOI:** 10.3390/nano12162838

**Published:** 2022-08-18

**Authors:** Linfeng Jin, Qinglin Pan, Xiaorui Li, Changqing Su, Zhongyu Wang, Haiying Wang, Lei Huang

**Affiliations:** 1 School of Materials Science and Engineering, Central South University, Changsha 410083, China; 2 School of Resources and Environment, Hunan University of Technology and Business, Changsha 410205, China; 3 School of Metallurgy and Environment, Central South University, Changsha 410083, China; 4 Chinese National Engineering Research Center for Control and Treatment of Heavy Metals Pollution, Changsha 410083, China; 5 School of Environmental Science and Engineering, Guangzhou University, Guangzhou 510006, China

**Keywords:** heavy metal, adsorption, nano adsorbents, melamine foam, assembly

## Abstract

Heavy metal pollution is a serious threat to human health and the ecological environment, but adsorption technology based on nano adsorbents can effectively treat the crisis. However, due to the nanoscale effect, nano adsorbents have some crucial shortcomings, such as recycling difficulty and the loss of nanoparticles, which seriously limit their application. The feasible assembly of nano adsorbents is an accessible technology in urgent need of a breakthrough. In this study, three-dimensional (3D) adsorbent (MF/Ti_3_C_2_T_x_/PmPD) with excellent performance and favorable recyclability was prepared by interfacial polymerization with melamine foam (MF) as the framework, two-dimensional (2D) titanium carbide (Ti_3_C_2_T_x_) as the bridge and Poly (m-Phenylenediamine) (PmPD) as the active nano component. The morphology, structure, mechanical property of MF/Ti_3_C_2_T_x_/PmPD and reference MF/PmPD were investigated through a scanning electron microscope (SEM), Fourier transformed infrared spectra (FT-IR), Raman scattering spectra and a pressure-stress test, respectively. Owning to the regulation of Ti_3_C_2_T_x_ on the morphology and structure of PmPD, MF/Ti_3_C_2_T_x_/PmPD showed excellent adsorption capacity (352.15 mg/g) and favorable cycling performance. R–P and pseudo-second-order kinetics models could well describe the adsorption phenomenon, indicating that the adsorption process involved a composite process of single-layer and multi-layer adsorption and was dominated by chemical adsorption. In this research, the preparation mechanism of MF/Ti_3_C_2_T_x_/PmPD and the adsorption process of Cr(VI) were systematically investigated, which provided a feasible approach for the feasible assembly and application of nano adsorbents in the environmental field.

## 1. Introduction

With the development of industry, heavy metal pollution events (such as Cr, Cd, Pb, As, Hg, TI, etc.) occur frequently, seriously threatening human health and the ecological environment [1,2,3]. In recent years, adsorption technology has been widely studied because of its simple operation and low cost [4,5]. Due to the advantages of the nanoscale effect, nano adsorbents have more active sites, a large specific surface area and high mass transfer efficiency, and show high adsorption performance for heavy metal [6,7]. Therefore, nano adsorbents (such as graphene, carbon nanotubes, biochar, polymer, biomass, layered bimetallic hydroxide and their derivatives) have been developed successively [8,9,10,11].

However, the nanoscale effect is a double-edged sword. Nano adsorbents are easy to lose and difficult to recover in the application process, which virtually increases the cost. Moreover, nano adsorbents that diffuse into the environment may cause potential harm to animal and plant safety. These defects are one of the important reasons why nano adsorbents are difficult to be widely employed at present. How to make full use of the high performance of nano adsorbents and overcome the shortcomings of nanoscale effect is a current challenge.

The assembly of nano adsorbents is an effective measure to overcome the defect of the nanoscale effect. Chen et al. prepared hydrogel-supported zero-valent iron adsorbent by ion exchange and in-situ reduction method, realizing the macro assembly of nano adsorbents [12]. Moreover, the adsorption performance of hydrogel-supported adsorbent was 11.6 times higher than that of the nano adsorbent. Safarzadeh et al. assembled cloisite 30B with polymeric hydrogel and applied it to removal cationic dyes, also showing efficient adsorption capacity [13]. However, the hydrogel process has some disadvantages, such as high energy consumption, high cost and complex reaction steps. In order to meet the practical applications, a cheap and simple assembly technology urgently needs to be developed.

Melamine foam (MF) has the advantages of light weight, high porosity, acid and alkali resistance, and high thermal stability [14,15,16]. In addition, MF is cheap and easy to obtain, and can be customized according to needs. It has been reported that using MF framework to assemble nano adsorbents, such as nano zero-valent iron, metal organic framework and polydopamine, a series of 3D assembly adsorbents for environmental treatment have been prepared and shown excellent performance [17,18,19]. However, these current reports only assemble the nano materials, and there is little in-depth discussion on the regulation of the morphology and structure of the assembled nano materials. The nanoscale properties of nano materials are limited to a certain extent in the assembly structure.

Poly (m-phenylenediamine) (PmPD) contains abundant amino groups, which are positively charged under acidic conditions and can adsorb the negatively charged heavy metals acid ion through electrostatic adsorption and chelation [20,21,22]. In addition, PmPD has the advantages of simple preparation, low solubility and mass synthesis [23,24]. In recent years, our team has made continuous reports on the treatment of heavy metal [25,26,27]. Unfortunately, as a typical nano adsorbent, PmPD also encountered a bottleneck in its application.

In order to solve the problem of the nanoscale effect in the application of nano adsorbents mentioned above, and further explore the morphology and structure regulation in the assembly process, 3D MF/Ti_3_C_2_T_x_/PmPD was prepared by interfacial polymerization. Herein, PmPD was investigated as a typical nano adsorbent, and MF was used as the framework for the assembly of PmPD. By introducing 2D Ti_3_C_2_T_x_ with high affinity with PmPD, we hoped to optimize the morphology and structure of MF/Ti_3_C_2_T_x_/PmPD, to give full play to the nanoscale advantages of PmPD [28]. Cr(VI) is a heavy metal that seriously threatens human health and ecological environment [29,30,31]. Therefore, MF/Ti_3_C_2_T_x_/PmPD was applied to the treatment of Cr(VI) in this paper, and the adsorption process was explored in detail. This research would provide a feasible approach for the assembly and application of nano adsorbents in the environmental field.

## 2. Material and Methods

### 2.1. Materials

MF with a size of 100 × 60 × 20 mm was purchased from the Shanghai Zhengyang foam Co., Ltd. (Shanghai, China), m-Phenylenediamine (mPD) (99.5%) was purchased from the Sinopharm Chemical Reagent Co., Ltd. (Shanghai, China), K_2_Cr_2_O_7_, Na_2_S_2_O_8_, NaOH, HCl and C_2_H_5_OH were analytical grade and purchased from Aladdin Reagent.

### 2.2. Preparation of MF/Ti_3_C_2_T_x_/PmPD

MF pretreatment: the MF was cut into 1×1×1 cm small cubes and put into ethanol for ultrasonic cleaning to remove impurities. Then, it was washed repeatedly with deionized (DI) water and dried in an oven at 60 °C for 24 h.

Preparation of the MF/Ti_3_C_2_T_x_/PmPD: First, 1 g mPD in 100 mL DI water was dissolved to obtain an mPD solution. Next,100 mL Ti_3_C_2_T_x_ solution (2 mg/mL) and ultrasonic were added for 20 min to evenly mix the mPD and Ti_3_C_2_T_x_ solution. Then, 20 mL Na_2_S_2_O_8_ solution was added to the above solution, where the molar ratio of Na_2_S_2_O_8_ to mPD is 1:1. After vigorous stirring at 800 rpm at 0 °C, the MF was quickly added and squeezed with tweezers. Then, after stirring the reaction system was kept at 0 °C for 12 h. After the reaction, the impurities were cleaned with DI water and freeze-dried for 12 h to obtain the MF/Ti_3_C_2_T_x_/PmPD. The preparation method of MF/PmPD was similar to that of MF/Ti_3_C_2_T_x_/PmPD, except that Ti_3_C_2_T_x_ solution was not added.

### 2.3. Characterization

The surface morphology and element distribution of 3D adsorbents were observed by SEM (FEI Nova NanoSEM 230, the gold spraying time was 180 s). The structure characteristics of 3D adsorbents were characterized by Raman scattering spectra (532 nm, Renishaw inVia, the scanning range was 500~4000 cm^−1^) and the FT-IR (Nicolet IS10; the scanning range was 500~4000 cm^−1^), respectively. The mechanical property of 3D adsorbents was tested by the universal testing machine (Instron 5982; the deformation variables were 30%, 50% and 70%, respectively).

### 2.4. Batch Experiments

Potassium dichromate (K_2_Cr_2_O_7_) was dissolved in DI water to obtain an aqueous solution with a certain concentration of Cr(VI). The 3D adsorbents were put into 20 mL Cr(VI) solution, then the mixture was shaken at 200 rpm at 25 °C for 8 h. The residual Cr(VI) concentration was detected by UV−vis spectrophotometer (540 nm). At 25 °C, 3D adsorbents were placed into 0.5 mol/L NaOH solution, sonicated for 30 min, shaken for 8 h, filtered and washed with DI water three times, and finally dried for the next adsorption. All the experimental data were the average values of three measurements, whose relative error was less than 5%.

Adsorption isotherms: the mathematical expressions of the Langmuir, Freundlich, Redlich–Peterson (R–P), Temkin and Dubinin-Radushkevich (D–R) models are depicted in Equations (1)–(5), respectively [32,33,34].

(1) $$q_{e}=\frac{K_{L}q_{m}C_{e}}{1+K_{L}C_{e}}$$

(2) $$q_{e}=K_{F}C_{e}^{\frac{1}{n}}$$

(3) $$q_{e}=\frac{{\mathrm{Kc}}_{e}}{1+{\alpha c}_{e}^{\beta }}$$

(4) $$q_{e}=\frac{\mathrm{RT}}{b\ln\left(K_{T}C_{e}\right)}$$

(5) $$\ln q_{e}=\ln q_{m}-{\beta \varepsilon }^{2}$$

$$\varepsilon =\mathrm{RT}\ln\left(1+\frac{1}{C_{e}}\right)$$

where q_e_ (mg/g) and c_e_ (mg/L) are the equilibrium adsorption capacity and equilibrium concentration of Cr(VI), respectively; q_m_ (mg/g) is the maximum adsorption amount of Cr(VI); K_L_ (L/mg), K_F_ (L/mg) and “n” are coefficients; “β” is an exponent between 1 and 0; α and K are the isotherm constants; K_T_ (L/mol) and B_1_ (J/mol) are the isotherm constants; R (J/(mol·K)) is the molar gas constant. When the E value was in the range of 1~8, 8~16 and >16 kJ/mol, it indicated that the adsorption process mainly involved physical process, ion exchange process and chemical adsorption process, respectively.

Adsorption kinetics: the mathematical expressions of pseudo-first-order, pseudo-second-order, Elovich, Intraparticle diffusion and Boyd adsorption models are depicted in Equations (6)–(10), respectively [35,36,37].

(6) $$\ln\left(q_{e}-q_{t}\right)=\ln q_{e}-k_{1}t$$

(7) $$\frac{t}{q_{t}}=\frac{1}{k_{2}q_{e}^{2}}+\frac{1}{q_{e}}t$$

(8) $$q_{t}=\frac{1}{\beta_{E}}\ln\left(\alpha_{E}\beta_{E}\right)+\frac{1}{\beta_{E}}\ln t$$

(9) $$q_{t}=k_{i}t^{0.5}+C$$

(10) $$B_{t}=-0.4977-\ln\left(1-F\right)$$

$$F=\frac{q_{t}}{q_{e}}$$

where q_t_ and q_e_ (mg g^−1^) are the adsorption amount of Cr(VI) at equilibrium and specific time, respectively; k_1_ (min^−1^) and k_2_ (g/mg·min) are the rate constants; α_E_ (mg/(g·min^2^), β_E_ (g/(mg·min), k_i_ (mg/(g·min^0.5^) and C (mg·g) are coefficients. 

## 3. Results and Discussion

### 3.1. Material Characterization

**Figure 1:**
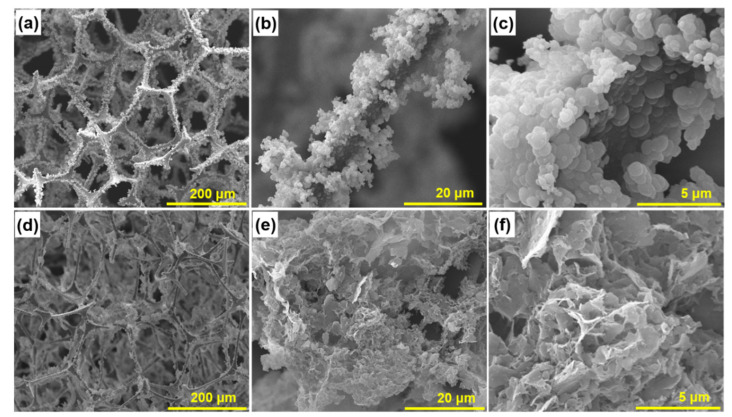
SEM images of MF/PmPD (**a**–**c**) and MF/Ti_3_C_2_T_x_/PmPD (**d**–**f**).

The morphology and structure of MF/PmPD and MF/Ti_3_C_2_T_x_/PmPD were observed by SEM. As shown in Figure 1a, MF provided an effective interface during the interfacial polymerization of PmPD, depositing PmPD nanoparticles in clusters. Moreover, MF had a developed network structure, which would provide an accessible site and ion transport path for the adsorption of heavy metals [38,39]. When the mPD contacted with the MF skeleton, the large π bond on the benzene ring of mPD formed a conjugation with π electrons of MF, which made mPD easy to enrich and induced interfacial polymerization [40,41]. When adding oxidant, the interfacial polymerization of mPD was triggered, and the deposition of PmPD nanoparticles on the MF skeleton was realized. However, the irregular accumulation of PmPD nanoparticles on the MF skeleton may reduce the specific surface area and mass transfer efficiency, which was not conducive to the improvement of the adsorption potential (Figure 1b,c) [26]. According to the previous research, Ti_3_C_2_T_x_ was 2D amazing material with a strong affinity to PmPD and other polymers, which could effectively optimize the morphology and structure, and then give full play to the nanoscale characteristics of PmPD [28,42,43]. As seen from Figure 1d–f, with the introduction of Ti_3_C_2_T_x_, the MF skeleton was covered by 2D snowflake Ti_3_C_2_T_x_/PmPD. It was revealed that the specific affinity between Ti_3_C_2_T_x_ and PmPD could adjust the morphology and structure of PmPD, and further strengthen the binding between Ti_3_C_2_T_x_/PmPD and MF. Compared with MF/PmPD, Ti_3_C_2_T_x_/PmPD on the surface of the MF skeleton was denser and adhered stably, which would help MF/Ti_3_C_2_T_x_/PmPD to express the nanoscale advantage in the treatment of heavy metals.

**Figure 2:**
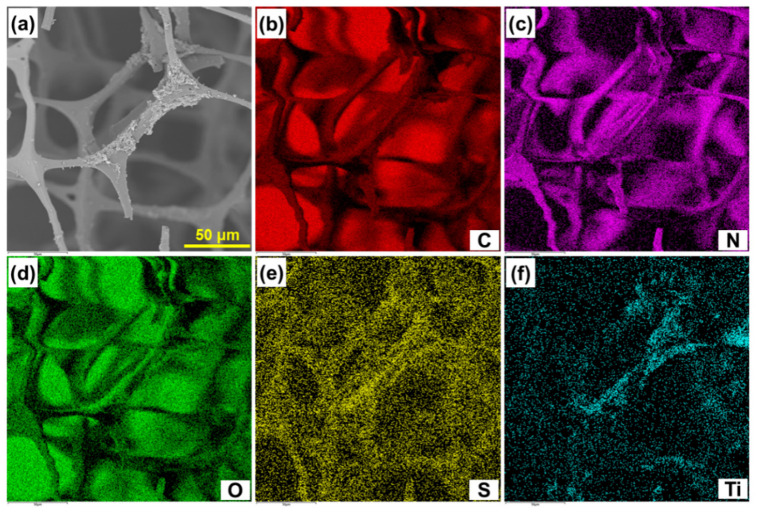
SEM image of MF/Ti_3_C_2_T_x_/PmPD (**a**); SEM-mapping images of C, N, O, S and Ti of MF/Ti_3_C_2_T_x_/PmPD, respectively (**b**–**f)**.

The element distribution of C, N, O, S and Ti was analyzed to judge the uniformity of Ti_3_C_2_T_x_/PmPD on the MF skeleton. The N element was from the MF and PmPD, the Ti element was from the Ti_3_C_2_T_x_, and the S element was from the Na_2_S_2_O_8_ in the oxidative polymerization process. As seen from Figure 2, Ti, N and S had obvious element distribution on the MF skeleton, indicating that the Ti_3_C_2_T_x_/PmPD realized the effective payload on the MF skeleton [44].

**Figure 3:**
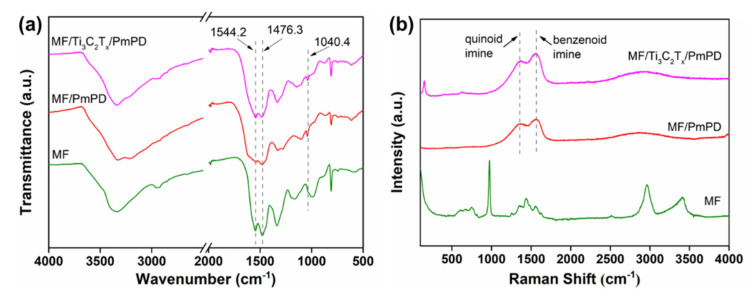
FT-IR (**a**) and Raman (**b**) spectra of MF, MF/PmPD and MF/Ti_3_C_2_T_x_/PmPD.

The structural characteristics of MF, MF/PmPD and MF/Ti_3_C_2_T_x_/PmPD were characterized by FT-IR, as shown in Figure 3a. For MF, the peak position at 3000~3500 cm^−1^ belonged to typical amino (-NH) stretching vibration. Methyl (-CH) bending vibration was located at ~1476.3, ~1335.9 and ~992.2 cm^−1^. Triazine cycloimino (C=N) stretching vibration was located at ~1544.2 cm^−1^. The peak position of carbonyl (C-O) corresponded to about 1160.4 cm^−1^. The bending vibration peak of the triazine ring was located at 810.4 cm^−1^ [45,46]. Interestingly, the FT-IR of MF/PmPD and MF/Ti_3_C_2_T_x_/PmPD were close to the superposition of the MF and PmPD or Ti_3_C_2_T_x_/PmPD, respectively. It should be noted that the peaks of functional groups contained in the MF basically exist, which indicated that the MF provided skeleton support while its own structure was not damaged. The complete skeleton structure endowed the MF/Ti_3_C_2_T_x_/PmPD with excellent mechanical properties.

Raman was used to further analyze the structural characteristics of MF, MF/PmPD and MF/Ti_3_C_2_T_x_/PmPD, as seen from Figure 3b. After loading PmPD or Ti_3_C_2_T_x_/PmPD, the Raman peaks of MF/PmPD and MF/Ti_3_C_2_T_x_/PmPD were the same as those of PmPD or Ti_3_C_2_T_x_/PmPD, and the corresponding quinone imine peaks and benzene secondary amine peaks were located at ~1364.56 and ~1549.1 cm^−1^, respectively [38]. After loading PmPD or Ti_3_C_2_T_x_/PmPD, the peak pattern of the MF weakened significantly. Because the Raman signal was detected by laser irradiation, the structure information of the surface of the detected object was mainly obtained. Therefore, for the MF/PmPD and MF/Ti_3_C_2_T_x_/PmPD, the Raman signal of the MF was shielded by PmPD or Ti_3_C_2_T_x_/PmPD to a certain extent. It indirectly explained the effective payload of PmPD or Ti_3_C_2_T_x_/PmPD on the MF skeleton.

**Figure 4:**
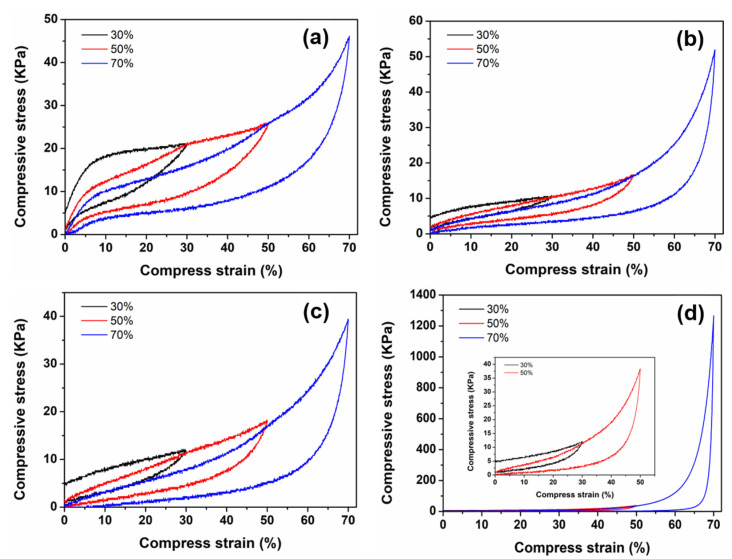
The stress-strain curves of the MF-T (**a**), MF (**b**), MF/PmPD (**c**) and MF/Ti_3_C_2_T_x_/PmPD (**d**) at different strains of 30%, 50% and 70%, respectively.

In practical application, the good mechanical property of adsorbents is conducive to improving its cycle stability. Hence, the mechanical property of MF-based adsorbents was tested [47]. Figure 4 showed the pressure stress curves of the MF before being treated (MF-T), MF, MF/PmPD and MF/Ti_3_C_2_T_x_/PmPD at the deformation variables of 30%, 50% and 70%, respectively. When the deformation of the MF-T pretreatment was 30%, 50% and 70%, the measured stress values were 21.13, 26.25 and 46.14 kPa, respectively, and the unloading curve could basically approach the origin. MF-T had no obvious plastic deformation, indicating that it had good mechanical performance [48]. After ethanol washing, repeated extrusion and other pretreatment processes, the corresponding stress values of the MF were 10.54, 16.64 and 51.90 kPa when the deformation was 30%, 50% and 70%, respectively. The stress value of pretreated MF in the low deformation region was lower than that of the MF-T, which may be caused by repeated and excessive extrusion during pretreatment, resulting in partial plastic deformation. However, the unloading curve of the MF was still close to the origin, indicating that the MF also had favorable mechanical properties.

After loading PmPD or Ti_3_C_2_T_x_/PmPD, the pressure stress curve of MF/PmPD and MF/Ti_3_C_2_T_x_/PmPD showed higher stress values in the low deformation region than MF, indicating that the mechanical properties of MF/PmPD and MF/Ti_3_C_2_T_x_/PmPD were significantly enhanced. Especially for MF/Ti_3_C_2_T_x_/PmPD, where the deformation was 70%, their stress values were increased to 342.29 and 1266.95 kPa, respectively. The huge improvement in mechanical property was due to the fact that 2D Ti_3_C_2_T_x_/PmPD was wrapped on the MF skeleton, which realized the synergistic reinforcement of the overall mechanical property.

**Figure 5:**
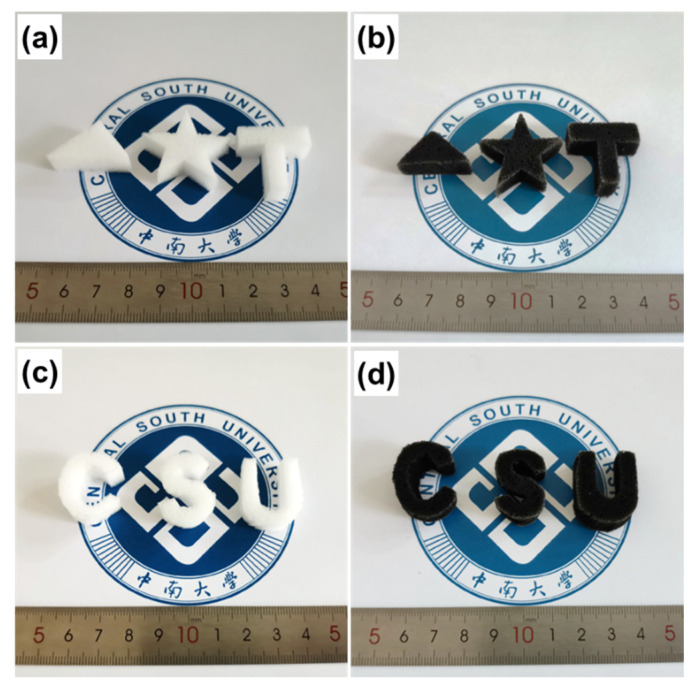
Different sizes and shapes of MF (**a**,**c**) and MF/Ti_3_C_2_T_x_/PmPD (**b**,**d**).

In addition to the good mechanical property mentioned above, MF-based adsorbents could be customized and cut into various shapes according to actual needs. Figure 5 shows a photograph of MF (a,b) and MF/Ti_3_C_2_T_x_/PmPD (c,d) with different shapes and sizes. This implies that MF/Ti_3_C_2_T_x_/PmPD could be applied to various complex pollution scenes.

**Figure 6:**
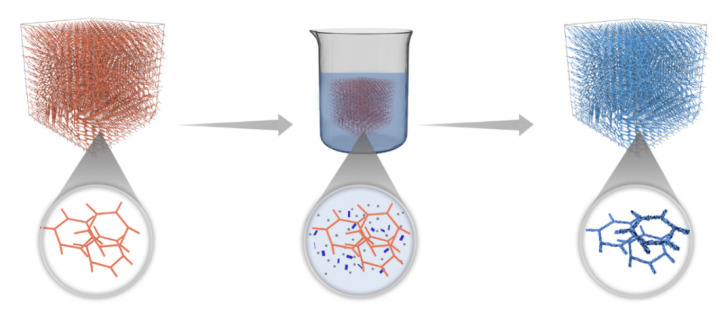
Preparation schematic diagram of MF/Ti_3_C_2_T_x_/PmPD.

Based on the above analysis, the preparation mechanism of MF/Ti_3_C_2_T_x_/PmPD could be inferred, as shown in Figure 6. First, the MF was cleaned and cut into a specific shape. mPD and Ti_3_C_2_T_x_ solution were mixed evenly. Due to the electrostatic interaction between mPD and Ti_3_C_2_T_x_, mPD was easy to preferentially enrich on the surface of Ti_3_C_2_T_x_, forming a Ti_3_C_2_T_x_ + mPD mixture [49,50]. Secondly, the oxidant was added to the above mixture in one measure and stirred vigorously to make the system solution and oxidant mix evenly in the shortest time possible. Then, the MF was quickly added and extruded several times, so that the network skeleton and pores of the MF were filled with the mixed solution (Ti_3_C_2_T_x_ + mPD + oxidant). Finally, the MF/Ti_3_C_2_T_x_/PmPD was obtained through interface polymerization, wherein the loading form of PmPD changed from 0D nanoparticles to a 2D snowflake.

### 3.2. Adsorption Experiments

**Figure 7:**
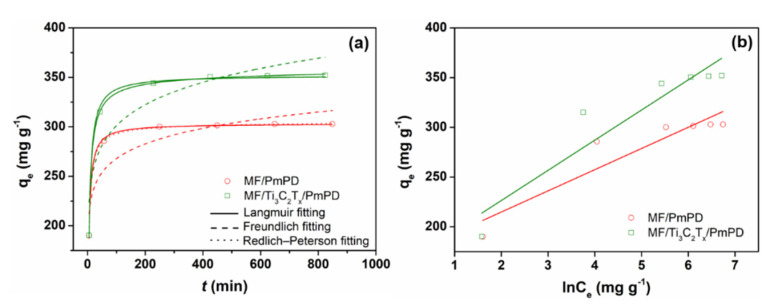
The fitting curves of Langmuir, Freundlich, R–P (**a**) and Temkin (**b**) models.

When the initial concentration of Cr(VI) was 100, 200, 400, 600, 800 and 1000 mg/L, the adsorption capacity of MF/PmPD and MF/Ti_3_C_2_T_x_/PmPD for Cr(VI) was investigated, respectively. The adsorption data were also fitted and analyzed by Langmuir, Freundlich, R–P, Temkin and D–R isothermal models, respectively. As shown in Figure 7, MF/PmPD and MF/Ti_3_C_2_T_x_/PmPD quickly reached the adsorption equilibrium in the low concentration region, showing excellent adsorption performance. As seen from Table 1, the correlation coefficients R^2^ of R–P were 0.9998 and 0.9997, respectively, which were higher than the R^2^ of the Langmuir, Freundlich and Temkin models, indicating that the adsorption of Cr(VI) involved the mixed adsorption process of a single layer and multi-layer [51,52]. In addition, the R^2^ of Langmuir were higher than those of Freundlich, revealing that the adsorption process was inclined to monolayer adsorption. The Langmuir maximum adsorption capacity of MF/PmPD and MF/Ti_3_C_2_T_x_/PmPD were 303.29 and 352.15 mg/g, respectively. The adsorption performance of MF/Ti_3_C_2_T_x_/PmPD was higher than that of MF/PmPD, which is attributed to the introduction of Ti_3_C_2_T_x_. Ti_3_C_2_T_x,_ that could adjust and optimize the morphology and structure of PmPD, so as to improve the mass transfer efficiency and adsorption performance of MF/Ti_3_C_2_T_x_/PmPD.

**Figure 8:**
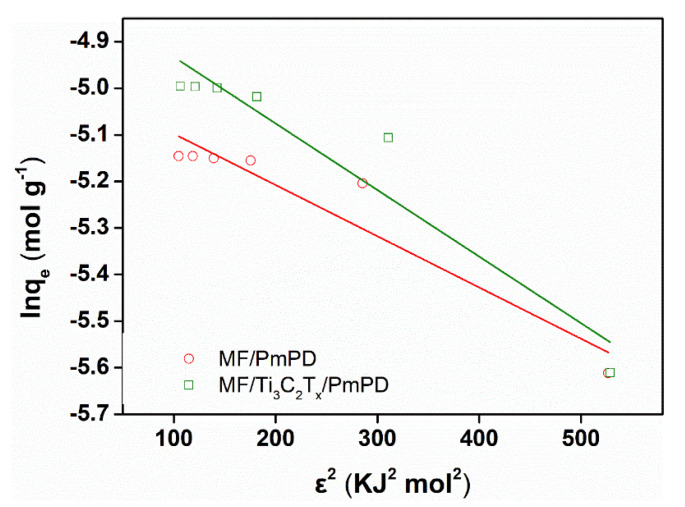
D–R isothermal adsorption fitting of Cr(VI) adsorption by MF/PmPD and MF/Ti_3_C_2_T_x_/PmPD.

The D–R model was also used to investigate whether the adsorption process of Cr(VI) was physical or chemical adsorption. The fitting line and the correlation coefficient were shown in Figure 8 and Table 2, respectively. The E value (kJ/mol) represents the average free energy, which could be obtained by fitting the D–R model. It can be seen from the table that the E values of MF/PmPD and MF/Ti_3_C_2_T_x_/PmPD were 21.32 and 18.7 kJ/mol, respectively, which were greater than 16 kJ/mol, suggesting that the adsorption process was dominated by chemical adsorption.

**Figure 9:**
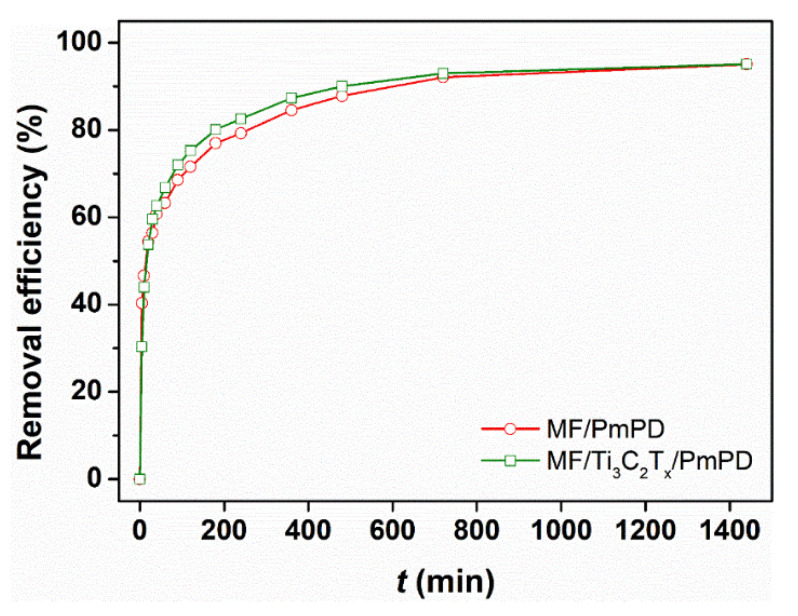
Effect of contact time on the Cr(VI) removal efficiency of MF/PmPD and MF/Ti_3_C_2_T_x_/PmPD (initial Cr(VI) concentration: 100 mg/L, dosage: 0.5 g/L, pH = 2, temperature: 25 °C).

When the initial concentration of Cr(VI) was 100 mg/L, the influence of time on the adsorption performance of MF/PmPD and MF/Ti_3_C_2_T_x_/PmPD for Cr(VI) was investigated, and the adsorption kinetic process was explored. As shown in Figure 9, MF/Ti_3_C_2_T_x_/PmPD had faster adsorption kinetics than MF/PmPD. This is due to the regulation and optimization of the morphology of PmPD by introducing Ti_3_C_2_T_x_, endowing MF/Ti_3_C_2_T_x_/PmPD with higher mass transfer efficiency [53]. At ~180 min, the removal rates of Cr(VI) by MF/Ti_3_C_2_T_x_/PmPD and MF/PmPD were 80.10% and 76.97%, respectively. After adsorption equilibrium, the removal rates of Cr(VI) by MF/Ti_3_C_2_T_x_/PmPD and MF/PmPD were 95.13% and 95.04%, respectively.

**Figure 10:**
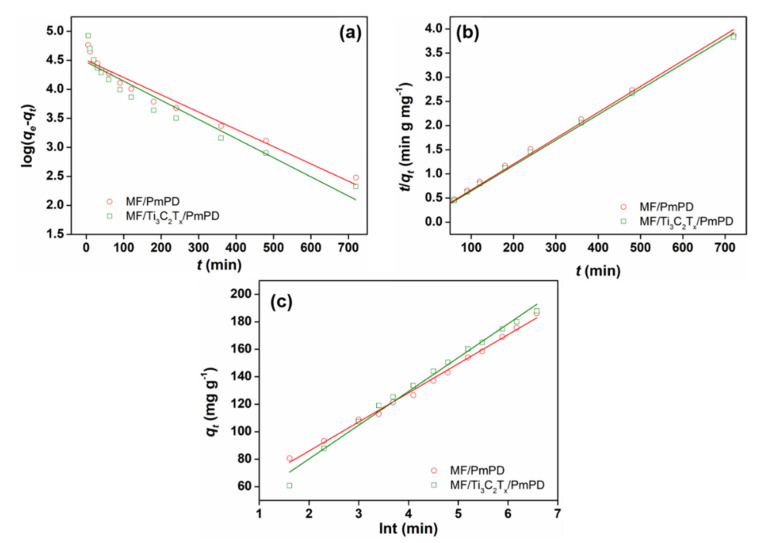
Linear fitting of pseudo-first-order (**a**), pseudo-second-order (**b**) and Enlovich (**c**) models of Cr(VI) adsorption by MF/PmPD and MF/Ti_3_C_2_T_x_/PmPD.

In order to systematically understand the dynamic adsorption process of Cr(VI) by MF/Ti_3_C_2_T_x_/PmPD and MF/PmPD, pseudo-first-order kinetic model, pseudo-second-order kinetic model and Enlovich model were applied to understand the adsorption process, as shown in Figure 10 and Table 3. Compared with the pseudo-first-order kinetic model and Enlovich model, the pseudo-second-order kinetics model of MF/Ti_3_C_2_T_x_/PmPD and MF/PmPD had a higher fitting coefficient, with R^2^ of 0.9979 and 0.9960, respectively, indicating that the adsorption process mainly involved chemical adsorption [54,55]. The fitting equilibrium adsorption capacities of MF/Ti_3_C_2_T_x_/PmPD and MF/PmPD were 189.75 and 186.92 mg/g, which were consistent with the actual measured values of 190.26 and 190.08 mg/g, respectively.

**Figure 11:**
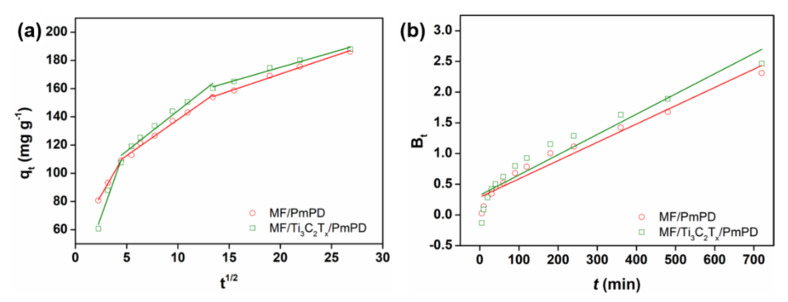
Linear fitting of intraparticle diffusion (**a**) and Boyd model (**b**) of Cr(VI) adsorption by MF/PmPD and MF/Ti_3_C_2_T_x_/PmPD.

The intraparticle diffusion model and Boyd model were used to investigate the key steps in limiting the adsorption rate in the adsorption process, as shown in Figure 11 and Table 4. MF/PmPD and MF/Ti_3_C_2_T_x_/PmPD contained three straight lines in the whole adsorption process, revealing that the adsorption process involved three different speed control steps (liquid film diffusion process, internal diffusion process and rapid adsorption equilibrium process) [56,57]. The adsorption properties of MF/PmPD and MF/Ti_3_C_2_T_x_/PmPD were based on the active component PmPD, so the adsorption kinetic process was generally consistent. For the internal diffusion linear fitting of MF/PmPD and MF/Ti_3_C_2_T_x_/PmPD, the fitting straight line in the second stage did not pass through the origin, indicating that the internal diffusion process was not the only key step to control the adsorption rate, and the adsorption process may also involve other influencing factors. The slope of the straight line fitted in the third stage was low, indicating that the adsorption process at this stage was close to the adsorption equilibrium stage. The boundary layer effect of adsorption materials usually had an important impact on the adsorption rate. The Boyd diffusion model of Cr(VI) adsorption by MF/PmPD and MF/Ti_3_C_2_T_x_/PmPD had a high linear fitting, and the fitting coefficients R^2^ were 0.9533 and 0.9284, respectively. The fitting lines had a large intercept, and the fitting lines did not pass through the origin, indicating that the control factors of the adsorption process mainly involved the internal diffusion and membrane diffusion processes.

**Figure 12:**
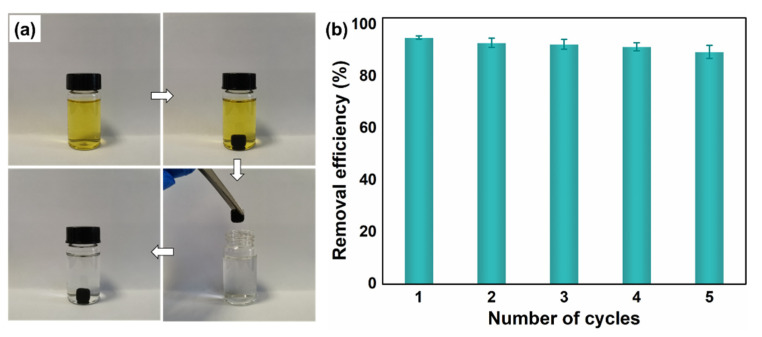
Schematic diagram of adsorption and separation of Cr(VI) by MF/Ti_3_C_2_T_x_/PmPD (**a**); MF/Ti_3_C_2_T_x_/PmPD cycle performance (**b**).

The 3D MF-based adsorbents not only had ideal adsorption performance and fast adsorption kinetics, but also showed significant advantages in the adsorption and separation, as shown in Figure 12. Taking MF/Ti_3_C_2_T_x_/PmPD as an example, after adsorbing the Cr(VI) solution with an initial concentration of 100 mg/L for 3 h, it can be seen that the Cr(VI) solution in the sample bottle had obviously changed from orange to a clear and transparent color, proving that a large amount of Cr(VI) had been effectively removed. After adsorption, MF/Ti_3_C_2_T_x_/PmPD could be separated quickly, and the removal efficiency still maintained above 90% after 5 cycles.

## 4. Conclusions

In order to improve the separability and heavy metal adsorption capacity of nano adsorbents, 3D MF/Ti_3_C_2_T_x_/PmPD was prepared by interfacial polymerization with MF as the framework, Ti_3_C_2_T_x_ as the bridge and PmPD as the active nano-adsorbent. Due to the regulation and optimization of Ti_3_C_2_T_x_ on the morphology and structure of PmPD, PmPD changed from 0D nanoparticles to a 2D snowflake. The adsorption capacity of MF/Ti_3_C_2_T_x_/PmPD for Cr(VI) reached 352.15 mg/g, and the 90% removal rate could still be maintained after five cycles, showing a good application prospect. The fitting of adsorption isotherm models and adsorption kinetic models showed that the adsorption process involved the mixed adsorption process of single-layer and multi-layer adsorption, as well as the chemical adsorption process. In this paper, the preparation mechanism of MF/Ti_3_C_2_T_x_/PmPD was investigated in detail, which provided a feasible approach for the assembly and application of nano adsorbents in the environmental field.

## Figures and Tables

**Table 1:** The correlation coefficient of Langmuir, Freundlich, R–P and Temkin models fitting.

Adsorbents	Langmuir			
	q_m_	K_L_		R^2^
MF/PmPD	303.29	0.37		0.9989
MF/Ti_3_C_2_T_x_/PmPD	352.15	0.24		0.9974
**Adsorbents**	**Freundlich**			
	**n**	**K_F_**		**R^2^**
MF/PmPD	12.86	187.29		0.7850
MF/Ti_3_C_2_T_x_/PmPD	10.19	191.61		0.8175
**Adsorbents**	**R–P model**			
	**K**	**α**	**β**	**R^2^**
MF/PmPD	106.61	0.36	0.99	0.9998
MF/Ti_3_C_2_T_x_/PmPD	89.84	0.27	0.99	0.9997
**Adsorbents**	**Temkin model**			
	**K_T_**	**B_1_**		**R^2^**
MF/PmPD	3241.58	21.31		0.8330
MF/Ti_3_C_2_T_x_/PmPD	236.00	30.34		0.8531

**Table 2:** Fitting parameters of D–R model of MF/PmPD and MF/Ti_3_C_2_T_x_/PmPD.

Adsorbents	q_m_ (mg/g)	β (mol^2^/kJ)	E (KJ/mol)	R^2^
MF/PmPD	356.67	0.11 × 10^−2^	21.32	0.9560
MF/Ti_3_C_2_T_x_/PmPD	432.30	0.14 × 10^−2^	18.70	0.8926

**Table 3:** Correlation coefficient of pseudo-first-order, pseudo-second-order and Enlovich kinetic models fitting.

Adsorbents	Pseudo-First-Order			Pseudo-Second-Order			Enlovich Model		
	q_e_	k_1_	R^2^	q_e_	k_2_	R^2^	α_E_	β_E_	R^2^
MF/PmPD	90.15	0.20 × 10^−2^	0.9553	186.92	0.22 × 10^−3^	0.9960	166.12	0.47 × 10^−1^	0.9952
MF/Ti_3_C_2_T_x_/PmPD	87.43	0.33 × 10^−2^	0.9115	189.75	0.23 × 10^−3^	0.9979	86.71	0.40 × 10^−1^	0.9881

**Table 4:** The fitted parameters of intraparticle diffusion model and Boyd model of Cr(VI) adsorption by MF/PmPD and MF/Ti_3_C_2_T_x_/PmPD.

	Adsorption Stage	MF/PmPD	MF/Ti_3_C_2_T_x_/PmPD
Stage 1	k_i,1_ (mg g^−1^ min^−0.5^)	12.58	20.53
	C_1_	52.89	17.83
	R^2^	0.9976	0.9265
Stage 2	k_i,2_ (mg g^−1^ min^−0.5^)	5.10	5.71
	C_2_	86.98	87.11
	R^2^	0.9888	0.9669
Stage 3	k_i,3_ (mg g^−1^ min^−0.5^)	2.44	2.10
	C_3_	121.52	133.10
	R^2^	0.9933	0.9803
Boyd model	Intercept	0.29	0.32
	R^2^	0.9553	0.9115

## Data Availability

Data sharing not applicable.
